# Medial prefrontal cortex (A32 and A25) projections in the common marmoset: a subcortical anterograde study

**DOI:** 10.1038/s41598-021-93819-z

**Published:** 2021-07-15

**Authors:** Jorge Alexander Ríos-Flórez, Ruthnaldo R. M. Lima, Paulo Leonardo A. G. Morais, Helder Henrique Alves de Medeiros, Jeferson Souza Cavalcante, Expedito S. Nascimento Junior

**Affiliations:** 1 Neuroanatomy Laboratory, Department of Morphology, Federal University of Rio Grande Do Norte, Natal, Brazil; 2 Laboratory of Experimental Neurology, the University of the State of Rio Grande Do Norte, Mossoro, Brazil; 3 Laboratory for Neurochemical Studies, Federal University of Rio Grande Do Norte, Natal, Brazil

**Keywords:** Immunochemistry, Structural biology, Biological techniques, Neuroscience, Cognitive neuroscience, Brain, Nervous system, Central nervous system, Brain

## Abstract

This study was aimed at establishing the subcorticals substrates of the cognitive and visceromotor circuits of the A32 and A25 cortices of the medial prefrontal cortex and their projections and interactions with subcortical complexes in the common marmoset monkey (*Callithrix jacchus*). The study was primarily restricted to the nuclei of the diencephalon and amygdala. The common marmoset is a neotropical primate of the new world, and the absence of telencephalic gyrus favors the mapping of neuronal fibers. The biotinylated dextran amine was employed as an anterograde tracer. There was an evident pattern of rostrocaudal distribution of fibers within the subcortical nuclei, with medial orientation. Considering this distribution, fibers originating from the A25 cortex were found to be more clustered in the diencephalon and amygdala than those originating in the A32 cortex. Most areas of the amygdala received fibers from both cortices. In the diencephalon, all regions received projections from the A32, while the A25 fibers were restricted to the thalamus, hypothalamus, and epithalamus at different densities. Precise deposits of neuronal tracers provided here may significantly contribute to expand our understanding of specific connectivity among the medial prefrontal cortex with limbic regions and diencephalic areas, key elements to the viscerocognitive process.

## Introduction

The cognitive-behavioral activity is integrated with the emotions in the supra-modal regions of the cerebral cortex. A wide range of behaviors is coordinated mainly by the action of neurobiological processes integrated into the prefrontal cortex, establishing neuronal connections with most of the cortico-subcortical areas of the central nervous system. Although the activity of this cortex is generally associated with the most elaborate functional processes of human behavior as well as non-human primates, it is the medial region (mPFC) where major connections bridging cognitive activity with the emotions are structured. These connections through their networks with subcortical structures^1^ that intervene in the decision making and behavior of the individual are constituted within the three definite areas of the limbic regions of the brain^2,3^: the anterior cingulate cortex (A24), the prelimbic (A32) and the infralimbic (A25). These regions are essential for maintaining the connectivity, integration, and modulation of these type of functional processes.

The mPFC is also essential for the detection of and attention towards novelties, contextual evaluation, executive function, and objective-directed behavior. This cortex presents a significant activity that intervene in different ways in the cognitive and emotional processes, modulated by their excitement and inhibition, and is related to visceral control, mechanisms of reward and motivation, and decision making. Besides cognition and behavioral control, brain imaging studies have implicated mPFC activity in the pathophysiology of mood and anxiety disorders, including post-traumatic stress disorder and major depressive disorder^2,4–6^. The mPFC is structurally heterogeneous. It has also been observed that the division of the cortices within the mPFC corresponds with distinct cytoarchitectonic areas, which had no evident differentiation in the granular layers II and IV, and that these areas have high cell density in layers III and V. Moreover, the supragranular layers are considerably thicker than the infragranular layers^7^. Thus, these areas would correspond to the A32 and A25 regions in the common marmoset, located in the mPFC caudal portion. These aspects were identified in the medial wall of the prefrontal cortex in monkeys. The A32 and A25 cortices are composed of a heterogeneous population of long-range pyramidal neurons that receive, integrate, and retransmit ascending information from subcortical origins^1^.

It was described that the neuronal circuits of the mPFC are distributed mainly to the A32 and A25 regions, which are correlated by compromises in the anterior cingulate circuit and more posterior subcortical regions^7–9^. It appears that "this region is an area of confluence of the cincture cortex, orbitofrontal region, and dorsolateral cortex"^2^^(pp.118)^. Thus, it is not surprising to note that patients with mPFC lesions exhibit severe social impairment and reduced behavioral flexibility^10–13^. A partial correlation between mPFC activity and a subset of neuropsychiatric disorders related to social behavior in humans led to the hypothesis that the excitation/inhibition balance in mPFC circuits might be critical to normal social behavior^14,15^. Taken together, several studies^16–24^ illustrate the idea that various pathways of adhesion and synaptic signaling that operate in the mPFC, as well as the structure and activity of subregions in this cortex, are critical in and contribute to the initiation, maintenance and behavioral modulation/regulation, and associated effective pathology.

Specifically, among these mPFC subregions, the A32 cortex would be involved in the evaluation of conflict and anxiety situations, in the expression of conditioned/learned fear (but not in its acquisition), in the search for rewards, and in the development of goal-oriented strategies^2,25–28^. The A25 cortex has been postulated as a regulator in the generation of emotional responses. A25 cortex participates in the inhibition and processing of negative emotions in the amygdala. It is also involved in habituation behaviors, stress responses, and memories of negative affect, being an important regulatory center of behavioral adaptation^2,6,24,29–32^. Furthermore, data suggesting functional differences between rodents and primates have been reported. Previous work in common marmoset, areas 25 and 32 would have causal, albeit opposite, functions in the regulation of behavioral correlates of negative emotion^33^; their activation or inactivation would lead to variations in autonomic and behavioral activity associated with negative emotion expectancies. We suggest that these primate areas differentially regulate negative emotion and symptomatology of affective disorders.

In general, it has been argued that the basic distinction between A32 and A25 cortex, relates to the functions of evaluation and behavioral regulation, respectively^2^. However, the specific mechanisms of the circuits mediating this activity are largely unknown. Nevertheless, one fact that could explain this opposing but coordinated functionality points to the fact that these two regions of the mPFC also establish reciprocal connections that influence the morphology and function of their global connectivity. Prominent and reciprocal projections of layers V and VI between A32 and A25 have been observed^34^. Specifically, the axons of projection neurons from A25 to A32, or from A32 to A25, terminate in layer VI and layer V, respectively. There is sufficient evidence to suggest that the A32 and A25 subregions have opposing but integrated influences on brain activity, particularly with activity associated with emotional processing behaviors^34–37^.

In summary, there were not data reveling the specific organization of mPFC pathways in primates. Previous studies are based on unprecise injections in the mPFC, which spread and contaminates many mPFC areas and layers, making impossible distinguish between circuits. The present work has solved this problem, highlighting these specific circuits separately in a primate specie.

## Results and discussion

### Connectivity in primates

**Figure 1:**
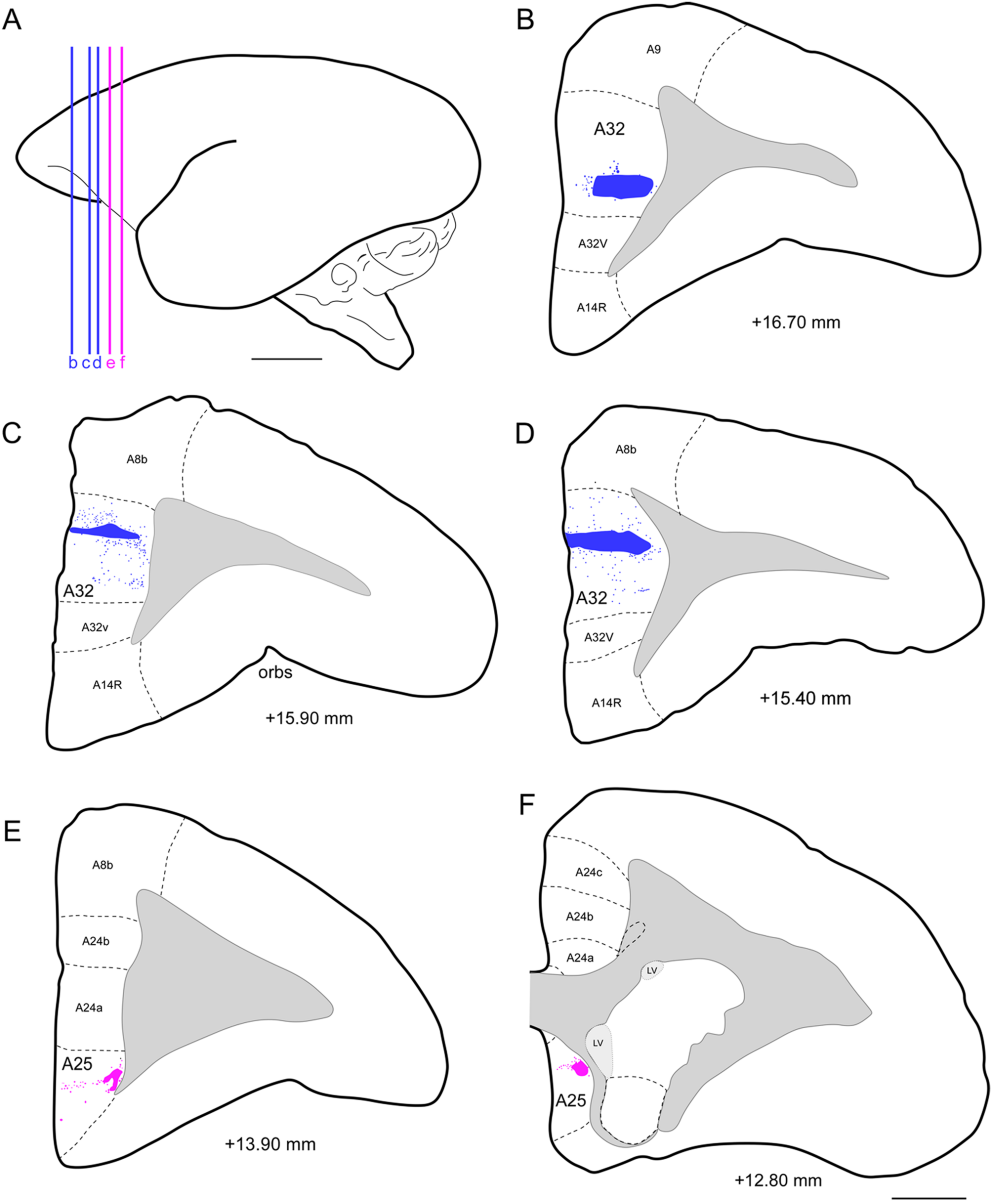
Injection centers and the location of the interaural levels (IA). (**A**) Interaural levels of the five cases in view A-P (marked with color lines); (**B**) (case 5), (**C**) (case 2), (**D**) (case 1): injections in A32 (blue); (**E**) (case 3) and (**F**) (case 4): injections in A25 (purple); IA level location based on the atlas “The marmoset brain in stereotaxic coordinates”); Scale bar (**A**) 5 mm; (**B**–**F**) 1000 µm. The figure was drawn in Canvas software version 12.0^76^.

**Figure 2:**
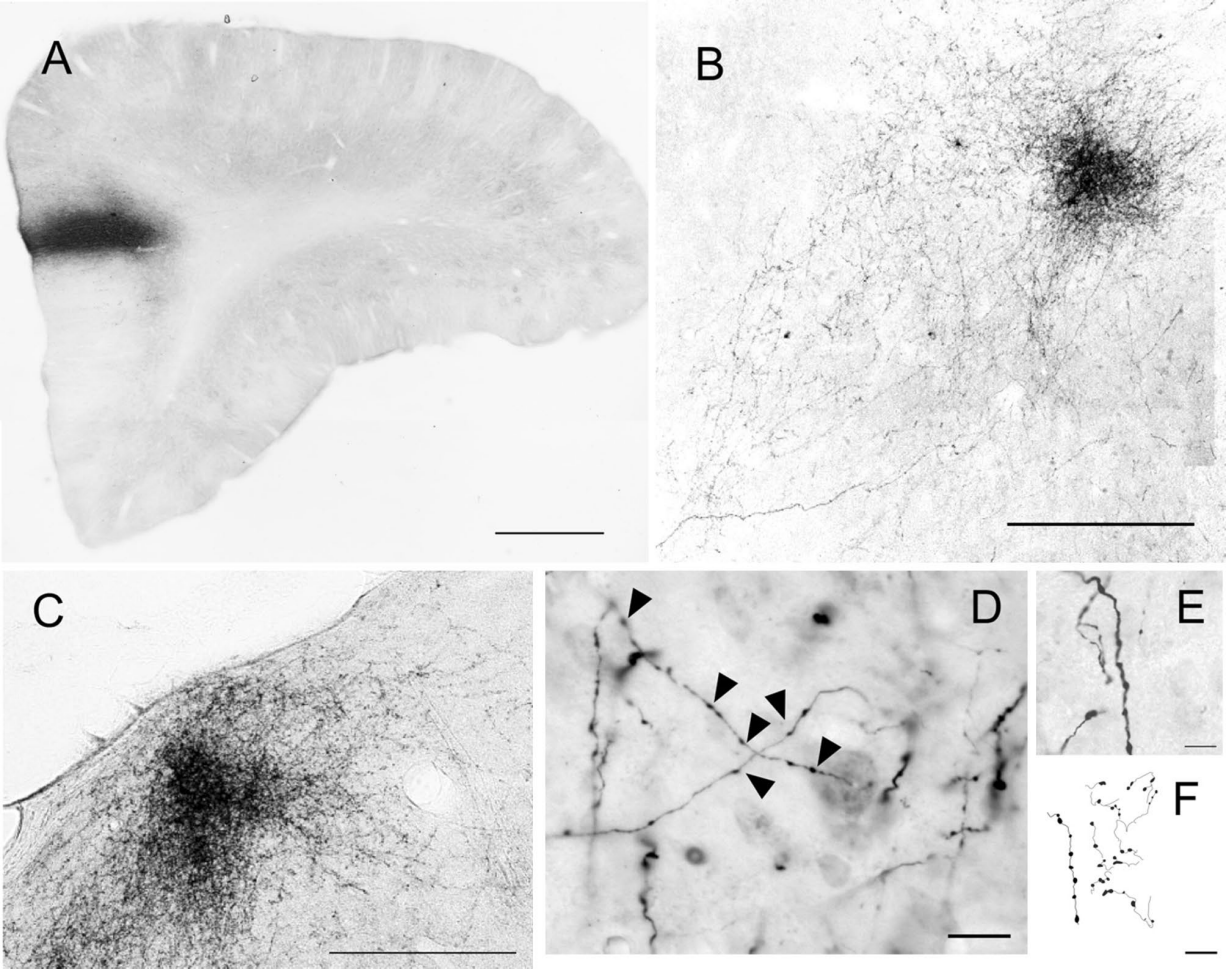
(**A**) Coronal section of the PFC, showing the center of injection of BDA in A32 (case 1); (**B**,**C**) Fiber-Plexus of BDA labeling in the MD thalamic nucleus; (**D**,**E**) higher magnification of fibers and varicosities; (**D**) Typical axonal fibers found in the diencephalic and amygdaloid complexes. The arrows signal those larger synaptic buttons that suggest possible synaptic terminals (although the fibers are similar in the two subcortical complexes, the sample image corresponds to fibers of the thalamus MD nucleus); (**F**) camera lucida draws of the BDA fibers; Scale bar (**A**) 1000 µm; (**B**,**C**) 200 µm; (**D**) 50 µm; (**E**,**F**) 10 µm. The figure was drawn in Canvas software version 12.0^76^.

A few studies on neuronal projections linked to the mPFC have been made in primates. The data from our anterograde tracing study in the common marmoset monkey provide novel insights in allowing us to determine, with greater specificity, which fibers from our injection centers in the A32 and A25 cortices (Figs. 1 and 2A) have been projected to different interaural levels (IA) within the diencephalic and amygdaloid regions. This enables us to clarify the distribution of fibers within the nuclei that constitute these subcortical areas. Regarding the type of neuronal fibers, it has been postulated that it is the terminal button, rather than the thickness of the axons that represents the distinguishing characteristic between type 1 and type 2 axons. Type 1 fibers are relatively uniform up to their terminal button. The type 2 fibers are characterized by presenting synaptic buttons along their length^38,39^. The characteristics of axonal fibers found in subcortical complexes of the common marmoset could be morphologically similar to type 2 modulating fibers (Fig. 2D–F). The size of the buttons could be an indicator of the possible establishment of synaptic connections wherein, hypothetically, larger diameter varicosities (signaled with arrows) could indicate the presence of organelles that intervene in the electrochemical exchange of the synaptic endings. Thus, this type of fiber, with varicosities lined along the largest axonal axis, were more frequently observed in our sample.

### Afferent projections from A32 and A25 primates

### Hypothalamus

**Figure 3:**
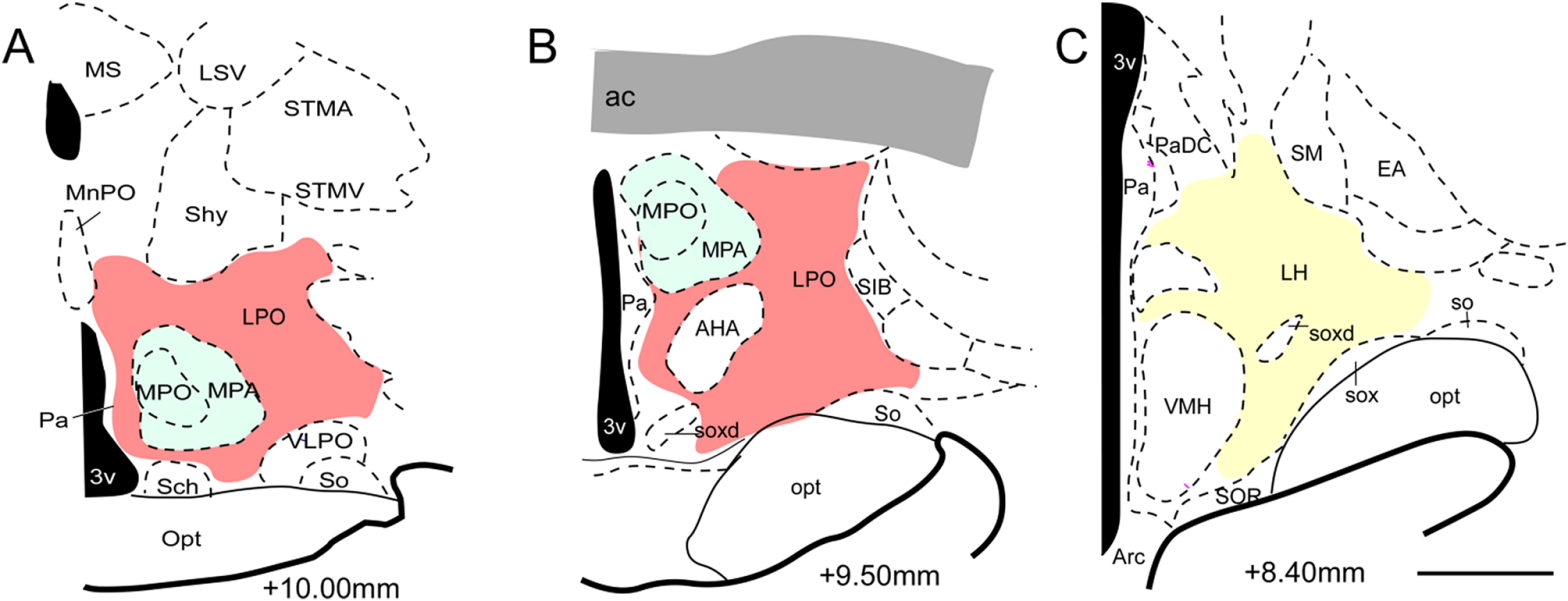
(**A**–**C**) Schematic draws representative coronal sections of the marmoset hypothalamus by regions in colors; Red: Lateral preoptic area; Yellow: Lateral hypothalamic area; Green: Medial preoptic area: Scale bar: 1000 µm. The figure was drawn in Canvas software version 12.0^76^.

Based on our research observations in the common marmoset, the hypothalamic complex (Fig. 3A–C) received fibers from two cortices (A32 and A25), covering its entire rostrocaudal extension, as already observed in the anterograde study of the mPFC in common marmoset monkey by Roberts et al.^7^.

**Figure 4:**
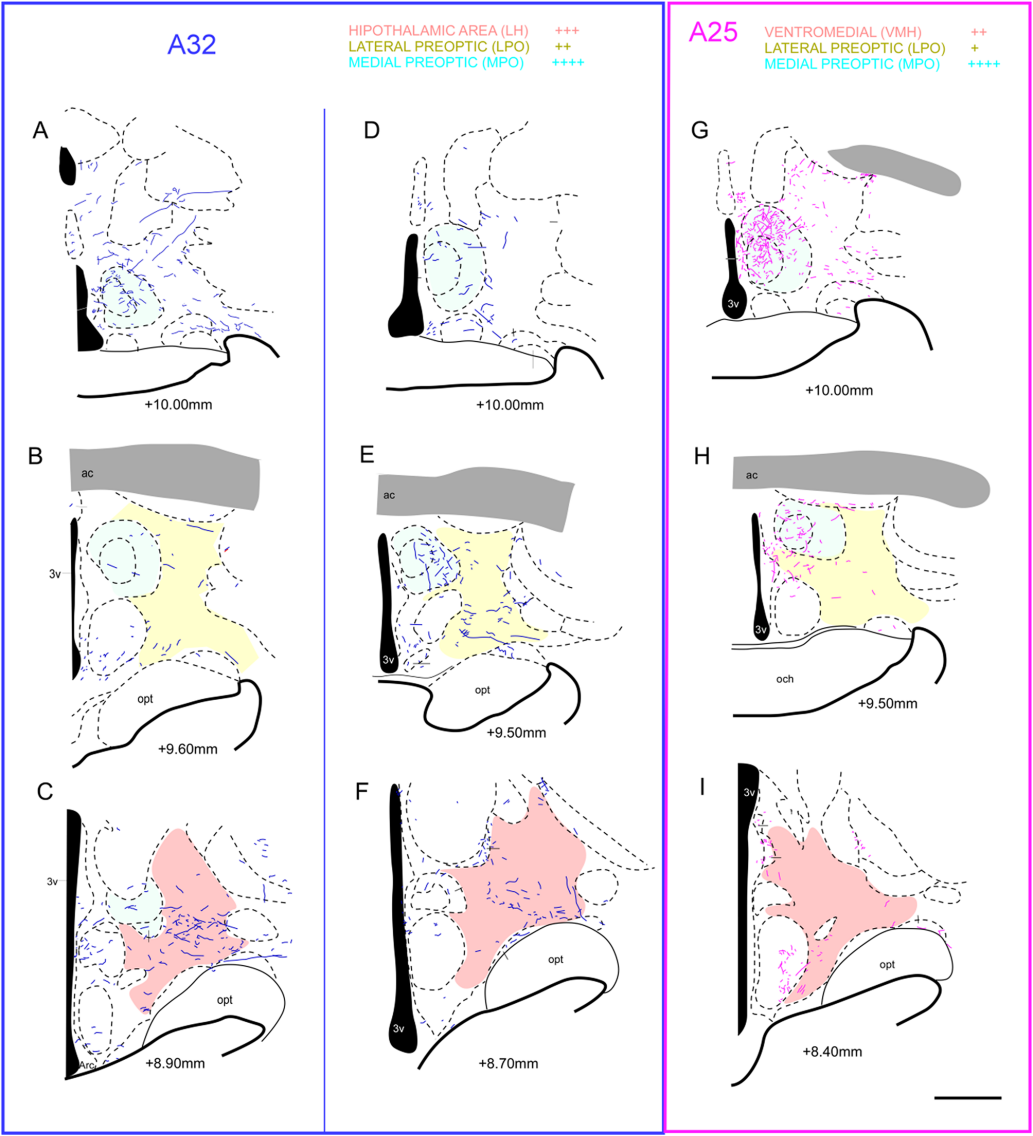
Location of the fibers from the A32 (blue) and A25 (purple) cortex in the hypothalamic complex. (**A**–**C**) case 1 (A32); (**D**–**F**) case 2 (A32); (**G**–**I**) case 3 (A25); Pink: Lateral preoptic area; Yellow: Lateral hypothalamic area; Green: Medial preoptic area; Scale bar: 1000* µm*. The figure was drawn in Canvas software version 12.0^76^.

The fibers originated in A25 presented a pattern of distribution and organization larger than those projected from the A32. The A32 directed the largest fiber cluster to lateral and posterior areas of the hypothalamic complex (Fig. 4A–F), while the A25 concentrated the large groups of fibers in areas and nuclei of more medial location (Fig. 4G–I). Specifically, both A32 and A25 directed fibers to Pa, MPO, MPA, and VMH. However, fibers emanating from the A32 presented higher density than those coming from A25, as was also found in the Japanese monkey^40^ at more rostral levels. Another study developed in common marmoset described that VMH did not receive projections from mPFC regions^7^. In turn, our observations reveal robust projections from the A32 to LPO (rostral), LH (caudal)^7^, MnPO, and AHA nuclei than the A25. Both cortices project vaguely to the SO. The fibers that reached the SHy, DM, AH, and Pe were exclusive to the A32, and those that reached the PaDC, PaLM, and JPLH were only from the A25. None of the cortices sent fibers to SCh^7^, sox, soxd, or SOR.

### Thalamus, epithalamus, and subthalamus

**Figure 5:**
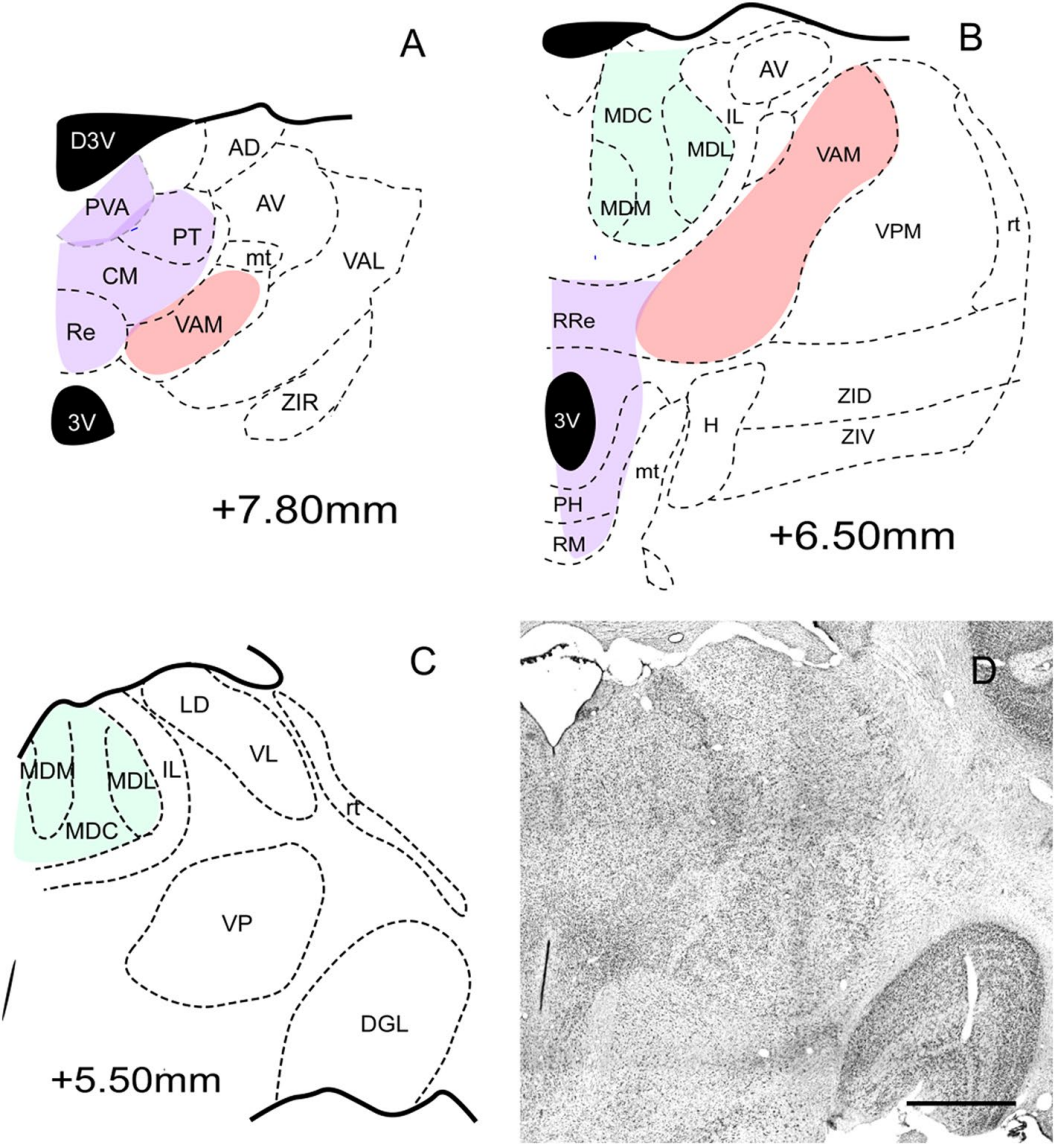
**(A–C)** Schematic draws representative coronal sections of the marmoset diencephalon; The main targets of A25 and A32 is represented by regions in colors**; **(**C**) Schematic draw based on coronal section of the Nissl presented in (**D**); Scale bar: 1000* µm*. The figure was drawn in Canvas software version 12.0^76^.

**Figure 6:**
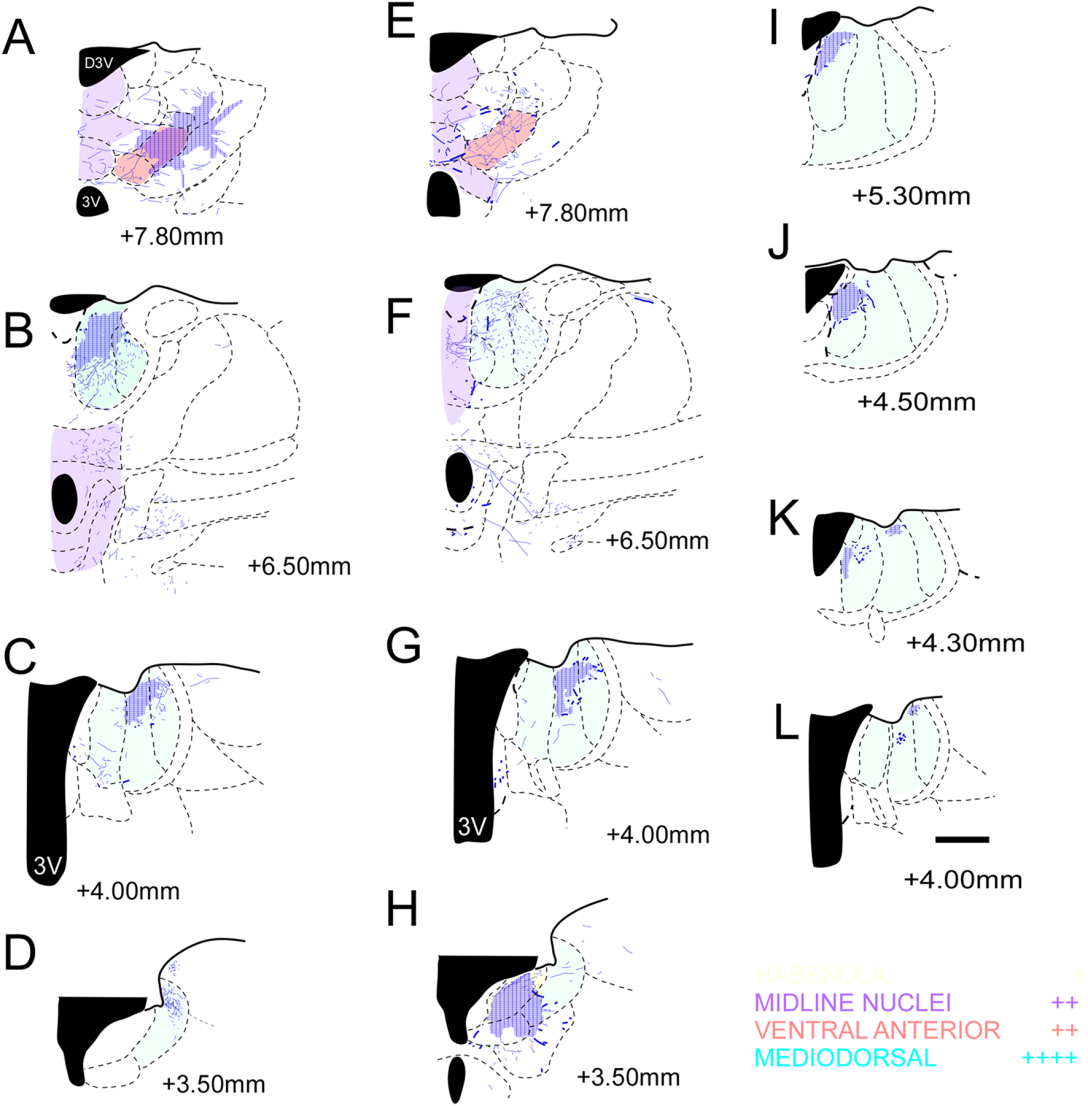
Location of the fibers from the A32 (blue) in the diencephalic complex. (**A**–**D**) case 1; (**E**–**H**): case 2; (**I**–**L**) case 5; The shaded area corresponds to the plexuses: high density of fibers. + : Isolated axons; +  + : Low presence of fibers; +  +  + : moderate presence of fibers; +  +  +  + : high presence of fibers; Scale bar: 1000 µm. The figure was drawn in Canvas software version 12.0^76^.

**Figure 7:**
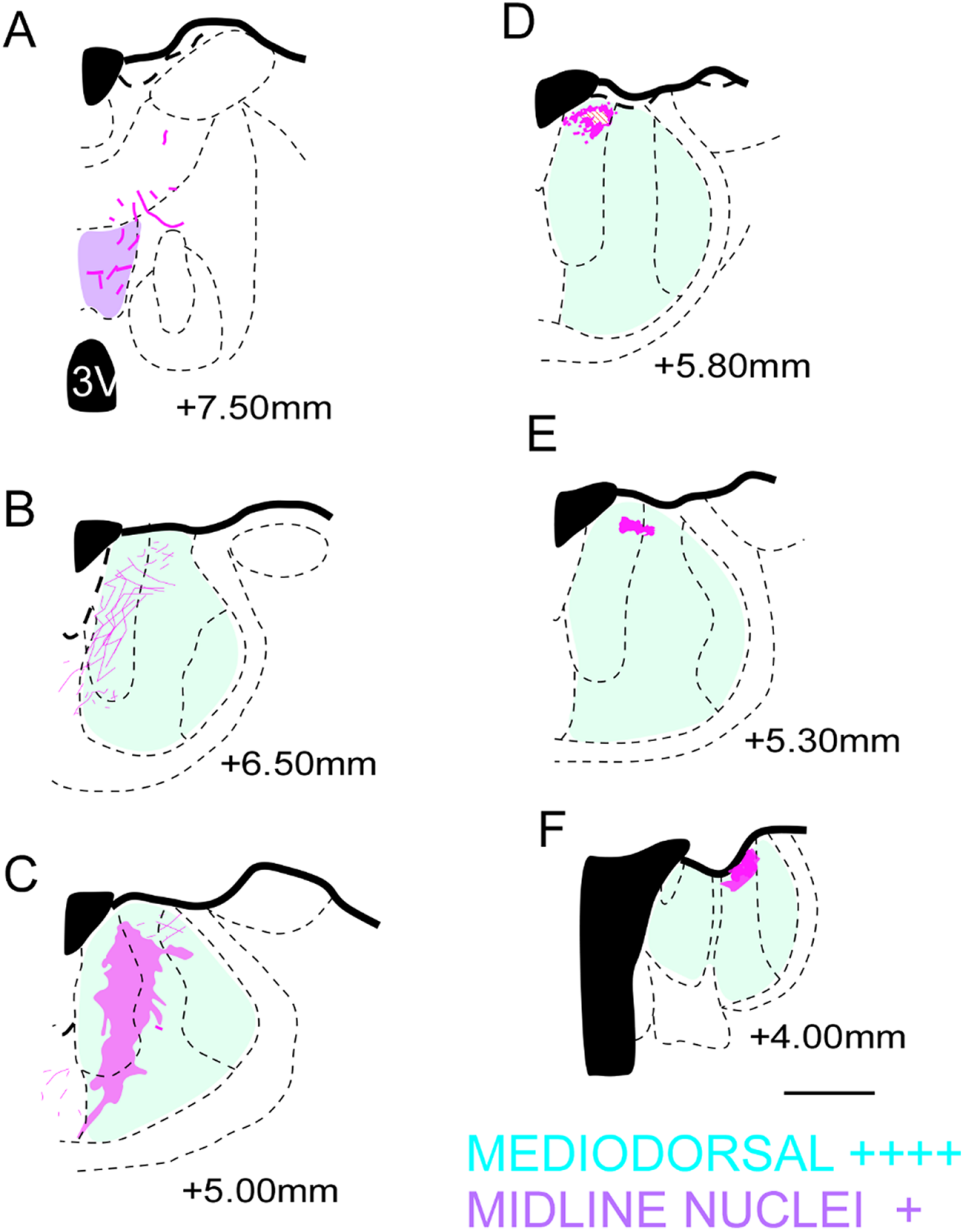
Location of the fibers from the A25 (in purple) in the diencephalic complex. (**A**–**C**) case 3; (**D**–**F**) case 4; The shaded area corresponds to the plexuses: high density of fibers. + : Isolated axons; +  + : Low presence of fibers; +  +  + : moderate presence of fibers; +  +  +  + : high presence of fibers; Scale bar: 1000 µm. The figure was drawn in Canvas software version 12.076.

Inside the thalamus of the common marmoset (Fig. 5A–D), we observed that both cortices -the A32 and A25—had the highest density of their fibers for the group of mediodorsal nucleus (MD). This was consistent with observations from earlier studies on Japanese monkey^40^ and common marmoset monkey^7^. Although only few fibers were observed in the nuclei in the earlier study with common marmoset^7^, in this study, we found that both the central MD (MDC) and medial (MDM) nuclei received fibers, especially, from the A32 and A25 cortices in sets of plexuses (fibers-plexus in Fig. 2B,C). The fibers originating from A25 were located more caudally in the lateral and medial portions of these nuclei (Fig. 6G–I), and those originating from A32 presented a more dorsal distribution (Fig. 5A–C). About the thalamic nuclei of the ventral region, we identified that the A25 cortex projected only for the VAM and we found that the A32 cortex projects a large group of fibers for all the subdivisions of the anterior ventral nucleus (VAM^40^, VAL, and VAMC). This was mainly in the rostral extension, with the caudal orientation^7^ being denser in VAM and VAL; moreover, the posterior levels were directed dorsally to MDC and its limits with MDM and lateral MD (MDL). We also observed that the projections for MDL were denser from A32 and smaller groupings coming from A25. In both cases, the location of the plexus was predominantly in the dorsal portion. The distribution of the A25 cortex fibers (Fig. 7A–F) was similar to the MD, while the dense plexuses were concentrated in the MDC and MDM subdivision (Fig. 7C), decreasing in the rostrocaudal direction (Fig. 7C,E–F). Although the projections for the midline nuclei (PV, PT, IMD, Re, and Rh) were weak^7^(Fig. 7A), we observed a greater number of fibers from the A32 that from A25, as was also demonstrated in the Japanese monkey^40^. We observed few fibers mainly in the PV (anterior PVA), PT, and Re (rostral with a caudal decrease); these fibers originated from the A32 cortex, while those of A25 origin were located exclusively in the Re and dorsal MD (Fig. 7D–F).

Within the anterior thalamic nuclei, moderate number of fibers were seen in A32 in the medial regions of AV and AD. From A25 to AM, we observed that the mPFC fibers for this group of nuclei were located mainly in AM and AV^7^. In the intralaminar nuclei we found that the A32 cortex sends fibers to the rostral group of intralaminar nuclei (CM, PC and CL), which was consistent with the results of other studies on common marmoset^7^. However, in the Japanese monkey^40^, it has been observed that the A32 does not extend projections to these nuclei, and that like our study, it has been found that the A25 projects moderately and exclusively to the CM (Fig. 6A,B,E,F). Conversely, we observed few fibers located dorsomedially within the MPul, with fibers projected exclusively from the A32 cortex (Fig. 6C,D,G,H). The studies on the Japanese monkey and the common marmoset found that fibers within the MPul are originated from the mPFC which are not restricted to the A32^7^ and that the A25 also projects to the MPul^40^.

With respect to the epithalamus, we observed that the habenular nuclei, mainly the lateral (LHb), received dense groups of fibers in the caudal portion from the A32 (Fig. 6H) and minimal amounts from A25 (in the rostral LHb). This was also described in the Japanese monkey^40^. Additionally, we observed that the A32 also projected to the basal portion of the medial habenula (MHb). Projections from the A32 to lateral and MHb nuclei were seen on the rostral surface, revealing a caudal orientation. In addition, the fiber density increases in plexus within the LHb and decreases in MHb (Fig. 6J–L). Likewise, only the A32 projected for the subthalamic areas in general and bordering areas, such as zona incerta (ZI), with a distribution of the fibers medially within them; Fig. 6B,F). However, previous studies on primates have not reported data on similar observations. These findings reveal and verify that the A32 projects densely within the diencephalon than within the A25 cortex.

### Amygdala

**Figure 8:**
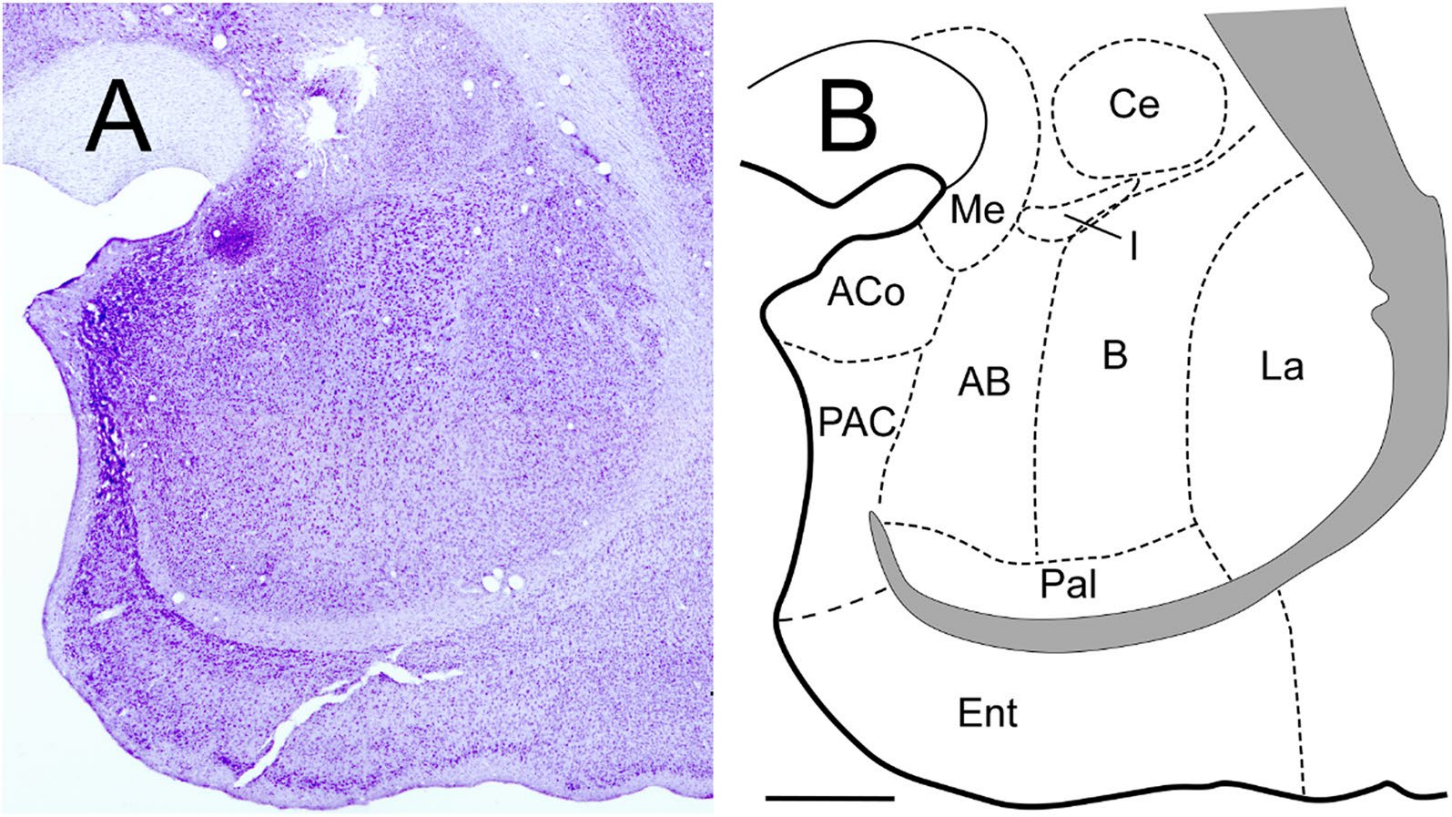
Amygdala of the marmoset (details in Morais et al.41). Coronal section of the amygdala at middle level (**A**) and schematic draw (**B**) of the amygdala nuclei. Scale bar: 1000 µm. The figure was drawn in Canvas software version 12.076.

**Figure 9:**
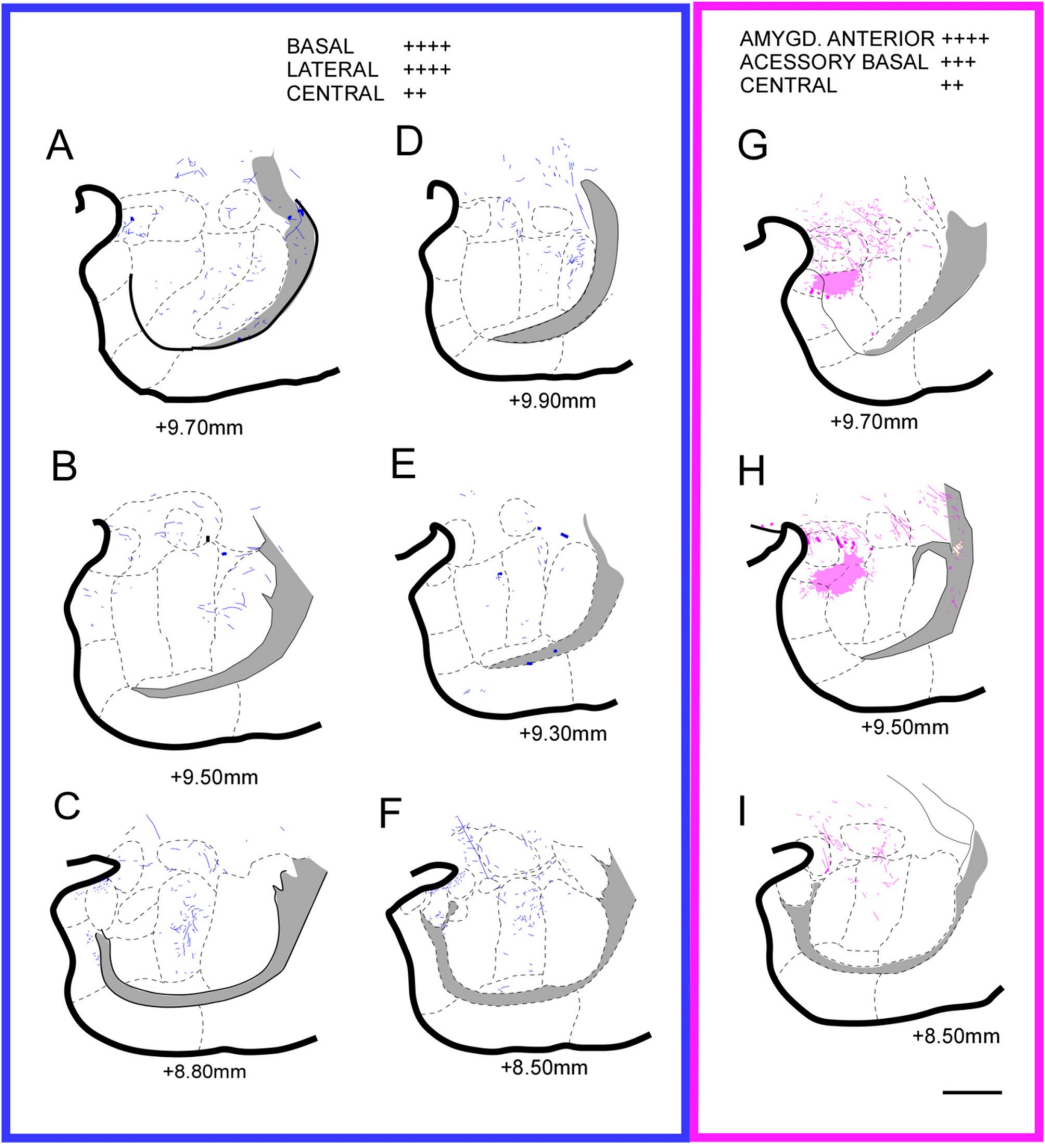
Location of the fibers from the A32 (blue) and A25 (purple) cortex in the amygdaloid complex. (**A**–**C**) case 1; (**D–F**) case 2; (**G–I**) case 3. The shaded area corresponds to the plexuses: high density of fibers. Scale bar: 1000* µm*. The figure was drawn in Canvas software version 12.0^76^.

Within the amygdala complex (Fig. 8A,B, details in Morais et al.^41^), we could determine that there were a specific distribution and organization pattern of the projected fibers from the A32 (Fig. 9A–F). In general, at rostral levels, the fibers are mostly concentrated in the Lateral nucleus (La). Caudally, the distribution of the largest grouping and fiber concentration is in the basal (B) and La. The A25 cortex also maintains a pattern of fiber distribution, which constitute plexus (Fig. 9G,H). Rostrally the plexus is in the upper half of the accessory basal nucleus (AB) and caudally remains in the B subdivision and extends to medial (Me) nucleus. However, towards the caudal region, the plexus disappears, and few fibers are in these areas. Previous studies have described the presence of fibers generally originating in the mPFC, reaching B area of the common marmoset^7^. The A32 and A25 cortices send fibers to AA, PCo, AB, and La, but those fibers from the A25 cortex are significantly denser than those from A32. A25 also sends more fibers to CeL than the A32. In turn, the A32 cortex projects more fibers to La than the A25 cortex. In La, fibers were almost exclusively from A32. Moreover, studies in the Japanese monkey^40^ and the common marmoset^7^ have described few fibers to the La nucleus. Further, in Me and I nuclei, are found groups of fibers in similar quantities, originating from A32 and A25. We observed that A32 and A25 direct fibers to central nucleus (Ce), in similar pattern to medial Ce. A25 projects more to lateral Ce, and central CeC only receives projections from A32^7^. The anterior cortical nucleus (ACo) received dense and exclusive projections from A25 (Fig. 9G,H).

In the middle region of the amygdala, within the B nucleus many fibers were observed^7^. We identified a plexus from A25 (Fig. 9G,H), and smaller groups of fibers from A32, as well as in the B caudal region, wherein A32 (Fig. 9C,F) projected more than A25 (Fig. 9I). Likewise, a dense group of fibers was directed to the AHi nucleus, coming exclusively from A32, as well as those found in the paralaminar (PaL). The A25 cortex did not project to these nuclei. Finally, the APir area, did not receive axons from any of the cortices A32 or A25. In general, the A25 cortex abundantly projects to the amygdaline nuclei than the A32. The results also support the data from classical research with anterograde tracers^42–44^, which is evidenced as an important source for the reception of prefrontal projections in the amygdala to the deep nuclei.

In a retrograde tracing study with Old World monkeys^45^, it was observed that there is connectivity between intermediate and magnocellular regions of the AB and B nuclei with the mPFC. As indicated earlier, our data pointed out anterograde marking from A32 and A25 in the mPFC to this region and the group of B nuclei (Fig. 9). This observation suggests the presence of reciprocal connections between these cortical and subcortical areas.

All the described data and results of the A32 and A25 projections for the subcortical complexes were summarized in Table 1.

**Table 1:** Summary of semiquantitative results on the appearance of fibers in subcortical areas/nuclei, discriminated by A**32** and A**25** cortices.

Area/nucleus	A32	A25
AA	+ +	+ + +
ACo	−	+ + + +
AD	+	−
AH	+	+
AHA	+	+
AHi	+	−
AM	−	+
AV	+ + + +	+ + + +
AB	+ +	+ + + +
B	+ + +	+ +
CeA	+	+ +
CL	+	
CM	+	+
DM	+	−
EA	+	−
H	+	−
H2	+ +	−
IMD	+	+
JPLH	−	+
La	+ +	+
LH	+ + +	+
LHb	+ + + +	−
LPO	+ +	+
MD	+ + + +	+ + + +
Me	+ +	+ +
MHb	+ + +	+
MnPO	+	+
MPA	+	+ + +
MPO	+ +	+ + +
MPul	+	+
Pa	+	+
PaL	−	+
PC	+	−
PCo	+	+
PSTh	+	−
PT	+	
PV	+	+
PVA	+	
Re	+	+
Rh	+	−
SCh	+	−
SHy	+	−
SO	+	+
SOR	+	−
sox	+	−
VA	+ + + +	+
ZID	+	−
ZIV	+	−

** + **: Isolated axons; + ** + **: Low presence of fibers; + ** +  + **: moderate presence of fibers; + ** +  +  + **: high presence of fibers.

### Common marmosets versus rodent’s connectivity

The studies developed in rodents (rats and mice, mainly), include the greatest variety of findings regarding cortical-subcortical connectivity interactions; thus, several classic and pioneering studies, describe projections from the mPFC to similar subcortical areas addressed in our study, albeit many differences could be reported. In marmosets, weak anterograde projections from A32 in the marmoset have been described in the Clastrum^46^. However, no fibers were visualized in the claustrum on the present work. Some technical considerations and differences could explain. In Reser et al. 2017, the BDA injection occupies the entire A32, including all cortical layers, and the ventral region of A8b. However, our study made microinjections in deep cortical layers (V and VI) in A32. These technical considerations could explain the presence of BDA labeling in the claustrum, considering that the superficial layers (2/3) could send projections to it.

### Hypothalamus

In rodents, it has been identified that the A32 and A25 cortices generate prominent projections to the hypothalamic nucleus LH, as well as to the medial region group of nuclei (MH)^3^. These results are consistent with findings in the marmoset. The results indicate that both mPFC cortices project to both the LH and the medial group nuclei. Although projections are dense from A32 and A25 to these nuclei, particularly A25 sends relativelymore fibers than A32 to the medial region nuclei, and A32 projects relatively more fibers than A25 to the lateral region of the hypothalamus. These results are similar to rodent connectivity. The rostral portion of the A32 and A25 projects to a major part of the rostrocaudal extent of the LH, and the caudal part of those cortices terminate in dorsomedial (DM) regions of the rat hypothalamus^47,48^. In marmoset, we identified that the A32 weakly projects to the DM, however, this nucleus did not receive projections from the injection sites in A25 cases.

Previous studies in rodents had vaguely reported that the A25 cortex has massive projections to the hypothalamus^49,50^, consistent with findings in marmoset. In rodents^6^ it was identified that these projections particularly reach the anterior nuclei, as well as the MPO, Pa, VMH, LH, and DM. Injections made in marmoset did not reveal projections from the A25 to the DM^6^. In retrograde tracing studies^51,52^, it has been identified that the LH projects to the A32 and A25 cortices, whereby it can be asserted that projections with the LH nucleus are reciprocal in rodents, which seems to be consistent with the findings in research developed in primates.

### Thalamus and epithalamus

Regarding the thalamic nuclei, the MD nucleus has been identified as the main receptor for efferences originating from the mPFC in rodents^3,6,53,54^. A fact that coincides with our results and the results in other primates studies^7,40^. In mouse^3^, the prevalence of projections from the A32 and A25 cortices has been described. Rostral areas of the A25 cortex distribute a higher concentration of fibers than the caudal one to the MD, as well as rostral region of the A32 cortex projects more densely (than the caudal-A32 portion) to this nucleus^54^, similar to the results obtained in marmoset. These connections are reciprocal; in rodents, the MD projects to the mPFC^55^; the MDM subdivision sends its fibers to the A25 cortex, while the MDL sends fibers to the A32^53^.

We found that the A32 and A25 cortices project densely to all subdivisions of the MD nucleus, with more abundant fibers originating from the A32 cortex. Fibers from the A32 reached preferentially the dorsal portion of the MDM, MDC, and MDL subdivisions, while the A25 distributed its fibers more laterally and medially within the subdivisions of the nucleus. Regardless of the fiber location within the MD, this nucleus received the highest fiber density projected from the mPFC cortices, both rodents^3,6,53,54^ and marmosets.

In marmoset, fibers were also found in the PVA, AM, IAM, Re, and the VA group. These results agree with data presented by several researchers that had rodents as experimental subject^6,54,56–59^. Particularly, in rodents it was identified^6^ that fibers in the PV originated in the A25 cortex, contrary to this, the fibers in this nucleus (anterior region—PVA) in marmoset came from the A32 and not A25, as a result of injections in these cortices. In rats, posterior PV nucleus, and not the anterior, is the receptor of projections from the mPFC, and the AV nucleus receives projections from the A32^54^, as in marmosets. Curiously, the A25 cortex also projected to anterior nuclei of the thalamus, albeit not to the AV, but the AM.

In mouse, fibers in the Re nucleus, are projected from the A25 cortex^60,61^. In our results in marmoset, fibers were identified from the A25 cortex to Re nucleus, as well as projections from the A32 cortex. In both cases, the fibers were located in the rostral portion of the Re. On the other hand, in rodent epithalamus region, it has been identified mPFC projections to the LHb nucleus^6^. These projections are probably originating in A32 and A25 cortices^62^. In marmoset, similar projections were found, although the A32 projected densely, while the A25 weakly sent its fibers to this habenular nucleus.

### Amygdala

Several studies covering connectivity interactions between the mPFC and the amygdala nuclei in rodents have identified the B nucleus as the main target of these cortical projections^1,6,34,54,63–66^; describing that the BL is densely innervated by A32 and A25 cortices. In marmoset, we identified that the BL receives fibers originating from the mPFC, although not densely.

Considering BL, our findings in marmoset are conflicting with data have been reported in rats. Such studies have been shown the presence of large fibers between A25-BL and smaller ones between A32-BL^67^ (Our results are opposite). The A25 of rodents provides moderate projections to BL^6^. From results of research that has inquired into connections originating in the amygdala, it is possible to say that the connectivity between A32 and A25 cortices to the BL nucleus is reciprocal. Rodent studies^1,3^ have reported the presence of fibers in the A25 and A32 cortices with the neuronal body within the BL area of the amygdala.

Evidence suggests that the dorsal portion of A32 cortex, and ventral division of A32/A25, innervate several amygdala nuclei in rats (Me, B, cortical nuclei, AA, CeC, CeM, and defined intercalated regions)^54,66^. In marmoset, we revealed both A32 and A25 cortices project equally to the Me and CeM nucleus, although CeL received fibers only from A32. The A25 cortex of marmoset sends fibers moderately to AA (more than A32). Regarding the nuclei of the cortical region (even when in mice it was not specified), we observed that marmoset A25 cortex projects moderately to ACo and PCo. In addition, PCo fibers were also observed coming from A32, although poorly. The dorsal B region was the nucleus that received densest projections from A25 cortex in the amygdala, with plexuses distributed rostrocaudal, and sparsely dense projections from A32.

In the marmoset, the A32 cortex projected a greater number of fibers to dorsal B, compared to A25, and a few to the ventral B. In rodents, studies do not report A25 cortex projections to the medial B^6^, however, they coincide in describing A25 fibers within the Me, and although in marmoset they are weak projections, in rat they show a considerable gradient with a rostrocaudal orientation^6^. In marmoset the A25 cortex projects fibers to CeM and CeL, but not to the CeC subdivision, as has been described in other rodent studies^6^. In marmoset, the CeC subdivision only received fibers from neurons in A32 (weakly).

In general, these comparison data between marmoset and rodents show consistency of projections from the A32 and A25 cortices of the mPFC to the regions of the diencephalon and amygdala in both animal study models. The existence of variations in the connectivity of these cortices varies when considering the orientation and distribution of fibers within the nuclei (rostral, caudal, dorsal, ventral), rather than the presence of fibers within them. Any lacunae in the empirical evidence are the result of a lack of studies, not a sustained lack of connectivity. These results only make an invitation for further research-oriented to deepen connectivity studies in primates, to compare with the abundance of results we have on rodent brain connectivity. In addition, these comparative descriptions, and the ample evidence of the connectivity of the mPFC, and its A32 and A25 cortices, provide support for the functional activity that has been attributed to these limbic cortices, as well as evidence of specific connectivity between the mPFC and subcortical regions, connections that have been described as essential for the evaluation and regulation of behavior and emotional responses.

### Final considerations

It is worth mentioning that the proximity between the anatomical analyses of neural networks in the common marmoset monkey and its macro-scale homology with the human brain preserves the relative positions of the primary sensory and motor areas, suggesting that the broad ordering and spatial relationships between networks of distributed associations might also be preserved^68^. These could be used to understand if the established networks are linked with their functioning in animal models. Likewise, owing to the functional characteristics of the subcortical regions that are approached, our results and observations could provide crucial evidence of the neuroanatomic substrates that intervene in the autonomous and endocrine function of the hypothalamus, and their modulation in interactions with the mPFC (A32 and A25) as fibers from this cortical region were found in the main nuclei that regulate these functions. Moreover, it is viable to highlight the apparent intervention of the mPFC (A32 and A25) in the functional activity of the amygdala as a part of the limbic regions in the modulation of emotions that guide and accompany behavior regulation being mediated by previous experience, according to the functional characteristics of the A32 and A25 cortices. The A32/A25-Thalamus connections represent a wide variety of functional influences, both sensitive and motor, on the cortico-subcortical activity, considering that the different nuclei of the thalamus are involved with specific and complementary functions in a way that they act as regulators and/or activators of brain activity. This was particularly evident when one studies the modulations in the subdivisions of the mPFC, as well as by their interaction with other subcortical centers of the encephalon in the consolidation and regulation of the activity that occurs in its afferent and efferent circuits. Therefore, it was suggested that the viscerosensory and visceromotor areas in the frontal lobes are in the agranular insular cortices, A32, and A25^40,69^. Therefore, these cortices were postulated as an autonomic region in the medial prefrontal cortex.

## Methods

### Research statement

All methods were carried out in accordance with relevant guidelines and regulations for animal research. All procedures were in accordance to the “ARRIVE Guidelines 2.0”^70^ and aproved by the Ethics Committee on the Use of Animals of the Federal University of Rio Grande do Norte, Brazil—CEUA/UFRN (authorization code: 024.028/2017). Also, we consulted the “Guidelines for the Euthanasia of Animals” of the American Veterinary Medical Association (AVMA)^71^ and the Brazilian Federal law on animal experimentation, the “Arouca law”^72^.

### Animals

The study was conducted on adult *Callithrix jacchus* primates (common marmoset monkey). Animals from both the sexes were utilized in the study. The common marmoset monkey is a neotropical primate that is not on the list of endangered species. Micro-injections of axonal markers (Tracers) were combined for both cerebral hemispheres in the same animal to minimize the number of subjects involved. The animals were obtained from the colony of the Nucleus of Primatology of the Federal University of Rio Grande do Norte, Brazil. The absence of telencephalic gyrus in the common marmoset's brain makes it possible to conduct a systematic study of serial sections of the entire brain. There is a growing consensus for data on cortical anatomy and physiology in this species; stereotactic atlases that are accurate and detailed for the common marmoset's brain (such as “The marmoset brain in stereotaxic coordinates”^73^ and “The brain of the common marmoset [*Callithrix jacchus*]: a stereotaxic atlas”^74^ have been considereably favorable for use. Eight experiments were performed on four animals, aged 2.5–3.5-year-old, and those with an approximate weight between 330 and 377 g. Despite the relative lissencephaly of the marmoset brain, few animals are enough to find the main similarities and differences of neuroanatomic structures and compare among several species of primates and humans^46^.

### Stereotactic coordinates and injection centers

The stereotactic coordinates were defined to reach the A32 and A25 cortices of the medial prefrontal cortex (Table 2), based on data from the atlas of the marmoset brain (The marmoset brain in stereotaxic coordinates^73^). In each animal, two experiments were performed (each hemisphere corresponds to 1 case). Using the same technique (iontophoresis), the first animal (animal 1) received an injection in the A32 cortex of both hemispheres, animal 2 in the A25 cortex of both hemispheres, and animals 3 and 4 in the A25 cortex of the left hemisphere and the A32 cortex of the right hemisphere.

**Table 2:** *Stereotactic coordinates of the injections performed with BDA in the common marmoset monkey cortex* (*Callithrix jacchus*); based on the atlas “The marmoset brain in stereotaxic coordinates”^73^. Coordinates in millimeters (mm); IontoPh.: Iontophoresis technique; time (`) in minutes.

**ANIMAL 1** (case 1)				**ANIMAL 1** (case 2)			
Hemisphere	Left	Lateral	+ 1.0	Hemisphere	Right	Lateral	+ 1.0
Area	A32	Dorsoventral	+ 3.6	Area	A32	Dorsoventral	+ 3.3
IontoPh. time	30`	Interaural	+ 15.40	IontoPh. time	25`	Interaural	+ 15.90
**ANIMAL 2** (case 3)				**ANIMAL 2** (case 6*)			
Hemisphere	Left	Lateral	+ 1.1	Hemisphere	Right	Lateral	+ 1.0
Area	A25	Dorsoventral	+ 6.7	Area	A25	Dorsoventral	+ 6.7
IontoPh. time	25`	Interaural	+ 13.90	IontoPh. time	25`	Interaural	+ 13.80
**ANIMAL 3** (case 4)				**ANIMAL 3** (case 7*)			
Hemisphere	Left	Lateral	+ 1.0	Hemisphere	Right	Lateral	+ 1.2
Area	A25	Dorsoventral	+ 6.5	Area	A32	Dorsoventral	+ 4.3
IontoPh. time	25`	Interaural	+ 12.80	IontoPh. time	15`	Interaural	+ 15.90
**ANIMAL 4** (case 8*)				**ANIMAL 4** (case 5)			
Hemisphere	Left	Lateral	+ 1.0	Hemisphere	Right	Lateral	+ 1.2
Area	A25	Dorsoventral	+ 6.7	Area	A32	Dorsoventral	+ 3.4
IontoPh. time	15`	Interaural	+ 13.90	IontoPh. time	25`	Interaural	+ 16.70

The analysis of the injection performed in Cases 6 and 8 revealed that there was no transport of the tracer. In Case 7 the analysis revealed that the injection was integrally deposited in another area of the PFC; therefore, only five cases are presented within the results.

### Neuronal tracer and injection technique

The tracer deposit was made through the iontophoresis injection technique. The choice of using one or the other technique was based on the characteristics and properties of the tracer and the ease of absorption in cell membranes^75^. Thus, iontophoresis deposits were performed, generating the opening of channels in cell membranes through the discharge of low frequency (500 *n*Amp) pulsed electrical current, which enabled the exchange of ions between the two cell environments. For marking the fibers and axonal buttons (synaptic terminal), the BDA anterograde tracer (Biotinylated Dextran Amine/Kit BDA-10,000. Ref. 7167, Fisher Lab, USA) was used.

### Stereotactic surgeries

At the time of taking the animals from the primatology center, they were administered a dose of Diazepam (1 mg/kg i.m.). Later, in the operating room, anesthesia was induced by inhalation of 3–4% of Isoflurane in pure oxygen. Further, a latex mask was placed on the face of the animal with a flow of 1–3% of Isoflurane in pure oxygen, which was administered to keep the animal breathing spontaneously throughout the procedure. Moreover, a 1 cc/h of saline solution was administered to the subcutaneous tissue to maintain adequate hydration.

The animals were positioned in the stereotactic instrument (Ref. Narishige model SN-2 N; Narishige Scientific Instrument Lab.) wherein they underwent a craniotomy of the frontal bone. After the removal of the dura mater layer, a tip of the micropipette (measuring between 15–20 μm in diameter) loaded with the BDA tracer, was introduced in the cortical areas of interest, according to the specific plane selected in the stereotactic coordinates of the atlas (Table 2). The procedure was conducted as per the technique of "injection" by iontophoresis whereby, a current of 500 *n*Amp pulsed (0.5 kW—7 secs; on/off) was downloaded using a generating source (Digital Midgard Precision Current Source Ref. 51,595) for 25–30 min. In conjunction with available reports, these procedure parameters allowed the marking of small groups of cortical cells. The micropipette was removed 5 min after the end of the iontophoretic application.

As soon as the "injections" were finished, the dura mater was occluded with *Spongostan* followed by the craniotomy and closed with dental acrylic resin. At the end of the surgery, the animals received an analgesic dose of *tramadol* (2 mg/kg sc.) as well as *Cafavecina* (CONVENIA, Pfizer), a broad-spectrum antibiotic, to confer protection against bacteria during the survival time of the animal. The time necessary to allow the proper passage of the tracer in the axon path was 15 days as calculated from the day after surgery. During this period, paracetamol was added to the water (5 mg/kg). As an anti-inflammatory drug, Ketoprofen (2 drops, vo.), was administered twice a day for one week.

### Tissue fixation procedures

After 15 days of the surgical procedure, the animals were removed from the primatology nucleus. During that time, the animals received a dose of Diazepam (1 mg/kg i.m.). In the perfusion room, the animals were euthanized by using an overdose of *Ketamine* (30 mg/kg i.p.) and *Xylazine* (1.5 mg/kg i.p.). The dosage of the drugs was three times to the recommended dosage for induction of anesthesia according to the protocols of the UFRN Ethics Committee-. As soon as the animals were euthanized, they were subjected to transcardiac perfusion, starting with 500 ml of saline serum (5 min; fast flow), followed by 1000 ml of paraformaldehyde solution in 0.1 M phosphate buffer (pH 7.4), with 2 cc of glutaraldehyde—added minutes before the perfusion (for 60 min; slow flow). Thereafter, 800 ml of a third solution containing paraformaldehyde in 0.1 M phosphate buffer (pH 7.4), and 15% of sucrose (for 60 min; slow flow) was used.

After perfusion, the bones of the cranial cap were removed, and the exposed brain was separated stereotactically. A single section was performed at the level of the interaural plane + 12.50 and the second section at the interaural plane -1.0 (middle level). Thus, the brains were divided into three (3) blocks: block one (1) containing the frontal portion of the brain, block two (2) having the entire temporal lobe and adjacent cortex, and block three (3) the occipital part of the cerebral hemispheres and the brain stem. The blocks were removed from the cranial cavity and immersed in the same fixation solution for 24 h (paraformaldehyde in 0.1 M phosphate buffer pH 7.4 to 4% and 15% sucrose); thereafter, the block was immersed in a phosphate buffer solution with 20% sucrose for three days. Subsequently, the sections were left in 30% phosphate buffer with sucrose until the moment of microtomy in the cryostat. All procedures related to surgeries and euthanasia were performed in the Neuroanatomy Laboratory of the Morphology Department at UFRN Biosciences Center.

### Microtomy and conservation of brain tissue

After remaining in the 30% solution of sucrose in phosphate buffer until they were submerged for cryoprotection, the blocks were positioned in the cryostat to perform the cryomicrotomy and obtain the histological sections. The equipment (Microtomy Cryostat—LEICA TEC. Ref. CM-1850) was calibrated and arranged for its operation according to its manual. Thus, it was programmed to make cuts of 50 µm. Each of the cuts was deposited in "vats" following a sequence of 1:7, in 0.1 M pH 7.4 phosphate buffer, until they were made available for the development of the corresponding histological techniques.

### Biotinylated dextran amine histochemistry

After the microtomy, the sections of the first series were mounted on previously gelatinized, dried, and dehydrated slides. One week later, they were processed to delimit the cortical centers and subcortical nuclei of interest in the common marmoset monkey by the Nissl staining method and then covered with a cover glass. In the third series of cuts, dextran-BDA (with glucose-oxidase) was revealed; sections were first rinsed for 20 min in oxygen peroxide to quench endogenous peroxidase and subsequently incubated overnight under continuous shaking in avidin–biotin-peroxidase complex (ABC; Vector Laboratories, Burlingame, CA) + diaminobenzidine (DAB) + 0,1% glucose-oxidase (Sigma-Aldrich) + 1% ammonium nickel sulfate in 0.1 M phosphate buffer pH. 7.4. A week later, these tissues were subjected to the procedure of dehydration and diaphanization for intensification with Osmium. Finally, the other series were stored in an antifreeze solution for possible repetitions and data confirmation, as well as other neuronal study techniques.

### Microscopy analysis and area delimitation

An entire series of Sects. (1:7) with BDA were examined by optical microscopy for the location of the injection centers and the images were superimposed on the tissue with Nissl marking to determine the location of the injection coordinates. Then we proceeded to locate the marked axonal branches (fibers) while tracing on a map the areas of interest of the brain hemisphere of the common marmoset monkey. The data was produced from the alignment of the contours of the coronal sections with the atlas of Paxinos et al.^73^ as a guide. This allowed us to have a global view of the subcortical distribution of fibers originating in the cells of the injection site. Similarly, representative sections of the content of the axons marked in the subcortical regions were identified and photographed using a lucid camera to reconstruct the subcortical pathways of axons and their distribution by area.

The second step involved a more detailed analysis of the topographic distribution pattern of the axonal branches in each area. Similarly, the arborizations of the fibers marked with the tracer were reconstructed from contiguous coronary sections. Also, in the third step, the coronal sections were reconstructed by drawing them and the fibers found in the subcortical complexes of interest were drawn one by one. This was done in their original location. All drawings and figures were made using the CANVAS software version 12.0^76^ and the tissue images were obtained in the lucid camera.

## Abbreviations

AA: Anterior amygdaloid area
AB: Accesory basal amygdaloid nucleus
ACC: Anterior cingulate cortex in mPFC
ACo: Anterior cortical amygdaloid nucleus
AD: Anterodorsal thalamic nucleus
AH: Anterior hypothalamic nucleus
AHA: Anterior hypothalamic area, anterior part
AHi: Amygdalohippocampal area
AM: Anteromedial thalamic nucleus
APir: Amygdalopiriform transition area
AV: Anteroventral thalamic nucleus
B: Basal amygdaloid nucleus
CeA: Central amygdaloid nuclei
CeC: Central amygdaloid nucleus, capsular part
CeL: Central amygdaloid nucleus, lateral part
CeM: Central amygdaloid nucleus, medial part
CL: Centrolateral thalamic nucleus
CM: Central medial thalamic nucleus
DM: Dorsomedial hypothalamic nucleus
EA: Extended amygdala
H: Nucleus of the H field of Forel
h2: H2 fasciculus (lenticular fasciculus)
I: Intercalated nuclei of the amygdala
IA: Interaural
IMD: Intermediodorsal thalamic nucleus
JPLH: Juxtaparaventricular part, lateral hypothalamus
La: Lateral amygdaloid nucleus
LH: Lateral hypothalamic area
LHb: Lateral habenular nucleus
LPO: Lateral preoptic area
MD: Mediodorsal thalamic nucleus
MDC: Mediodorsal thalamic nucleus, central part
MDL: Mediodorsal thalamic nucleus, lateral part
MDM: Mediodorsal thalamic nucleus, medial part
Me: Medial amygdaloid nucleus
Mgc: Magnocellular
MHb: Medial habenular nucleus
MnPO: Median preoptic nucleus
MPA: Medial preoptic area
MPO: Medial preoptic nucleus
MPul: Medial pulvinar
mt: Mamillothalamic tract
Pa: Paraventricular hypothalamic nucleus
PaDC: Paraventricular hypothalamic nucleus, dorsal cap
PaL: Paralaminar amygdaloid nucleus
PaLM: Pa hypothalamic nucleus, lateral Mgc. part
PC: Paracentral thalamic nucleus
PCo: Posterior cortical amygdaloid nucleus
Pe: Periventricular hypothalamic nucleus
Pir: Piriform cortex
PSTh: Parasubthalamic nucleus
PT: Paratenial thalamic nucleus
PV: Paraventricular thalamic nucleus
PVA: Paraventricular thalamic nucleus, anterior part
Re: Reuniens thalamic nucleus
Rh: Rhomboid thalamic nucleus
SCh: Suprachiasmatic nucleus
SHy: Septohypothalamic nucleus
SO: Supraoptic nucleus
SOR: Supraoptic nucleus, retrochiasmatic part
sox: Supraoptic decussation
soxd: Supraoptic decussation, dorsal part
VA: Ventral anterior thalamic nucleus
VAL: Ventral anterior thalamic nucleus, lateral part
VAM: Ventral anterior thalamic nucleus, medial part
VAMC: Ventral anterior thalamic nucleus, Mgc. part
VMH: Ventromedial hypothalamic nucleus
ZIA: Zona innominate amigdaloid
ZID: Zona incerta, dorsal part
ZIV: Zona incerta, ventral part
